# The quantified analysis of the correlation between medical humanities curriculums and medical students’ performance

**DOI:** 10.1186/s12909-023-04073-y

**Published:** 2023-08-11

**Authors:** Shiau‑Shian Huang, Chao-Chung Ho, Yeong-Ruey Chu, Jr-Wei Wu, Ying-Ying Yang

**Affiliations:** 1 Department of Medical Education, Taipei Veterans General Hospital, Taipei City, Taiwan; 2 College of Medicine, National Yang Ming Chiao Tung University, Taipei, Taiwan; 3 Bali Psychiatric Center, Ministry of Health and Welfare, Taipei, Taiwan; 4 Department of Public Health, China Medical University, Taichung, Taiwan; 5 Clinical Innovation Center, Taipei Veterans General Hospital, Taipei, Taiwan

**Keywords:** Medical humanities, Curriculums, Clerkship performance, Weighted average mark

## Abstract

**Background:**

A high-quality medical humanities (MH) education program is essential to developing a successful medical practitioner and can influence clinical performance. It is also vital to improve the evaluation of MH education to restore harmonious mutual relationships in medical care. However, studies have yet to discuss the correlation between the learning quality and quantity of medical humanities curriculums (MHC) and medical students' scores of clinical curriculums and clinical performance. The study aimed to assess the correlation between the learning quality and quantity of MHC and medical students' performance.

**Methods:**

We conducted a retrospective cohort study by analyzing a dataset of students' learning records. After excluding students with missing demographic information (*n* = 1) and overseas Chinese students (*n* = 15), the study included six- and seven-year program medical school students (*n* = 354) at National Yang-Ming University who were admitted between 2012 and 2014. The correlation between learning quality and quantity in MHC and students' following performance was evaluated by multivariable-adjusted regression analyses.

**Results:**

After adjusting for potential confounders (gender, residential area, age at enrollment, type of administration, and school program), the number of MHC with good learning outcomes was significantly correlated with clinical curriculum scores (*p* < 0.05), clerkship performance (*p* < 0.001), and weighted average mark (*p* < 0.001).

**Conclusions:**

Our study found a correlation between MHC with good learning outcomes and medical students' following performance. A future study of improving the quality of MH education is warranted.

**Supplementary Information:**

The online version contains supplementary material available at 10.1186/s12909-023-04073-y.

## Background

In the 1920s, Dr. Francis Peabody noted that "young graduates have been taught a great deal about the mechanism of disease, but very little about the practice of medicine or, to put it more bluntly, they are too scientific and do not know how to take care of patients [1]." Indeed, it has been recognized that physicians in training require more than just an understanding of scientific principles to become successful doctors [2]. An Australian study revealed that interns who had entered medical school with a mixed background (science and humanities) performed better in internships than those with a science-only background [3]. Thus, it is insufficient to possess only medical knowledge and technical skills to become a successful physician. Instead, we must also ensure that students become scholarly, compassionate professionals who collaborate well, communicate effectively, and advocate for individual patients and systems change [4, 5].

The medical humanities (MH) first developed as a distinct field of study in the USA, where the term was coined in 1948 [6]. The inclusion of the humanities in medical education may offer significant potential benefits to future physicians and the medical community [7]. Therefore, the cultivation of the humanistic aspects of medicine has received greater attention in recent decades. However, due to the lack of a consensus about the definition and precise role of the humanities in medical education [7], there is a dearth of empirically supported data showing that knowledge of the MH can predict student performance after admission to a medical program.

In Taiwan, modern MH education originated from the US National Committee on Foreign Medical Education and Accreditation in 1998, which evaluated Taiwan's medical education and deemed it non-comparable with that of the United States [8]. Taiwan's Ministry of Education then established the Taiwan Medical Accreditation Council to promote medical education reform throughout Taiwan, which further emphasize education reform in medical humanities, aimed at making students "first good citizens, then decent physicians [8]."

Although the MH is crucially important in medical education, few studies have discussed the correlation between the learning quality and quantity of medical humanities curriculums (MHC) and medical students' scores and clinical performance [9]. A review done in 2021 showed that many papers made only generalized statements about enhancing students' knowledge and skills [9]. Still, most articles did not cohesively present specific learning outcomes [9]. Nevertheless, a study done at Harvard University found a statistically significant association between Patient-Doctor I (the first-year MHC) assessment scores and clinical performance, including manual skills and humanistic and interactive student–patient ability scores [10]. Hence, the purpose of the study is to fill the information gap by attempting to "measure the immeasurable, " i.e., we created a study to assess the correlation between the learning quality and quantity of MHC and medical students' following performance with more generalized and objective methods.

## Methods

### MH in Taiwan

In Taiwan, the medical program consists of a four-year preclinical phase (two years of liberal education and basic medical sciences and two years of clinical curriculums) followed by a three-year or two-year clinical phase (clerkship) since the medical school program was reduced from seven to six years in 2013 [11]. Taiwanese medical students in clerkship are equivalent to 3rd or 4th-year medical students undergoing clinical training in four-year graduate medical programs in Western countries [12]. In the first and second years of medical school, curriculums related to MH were strengthened to meet the six core competencies of the Accreditation Council for Graduate Medical Education [13]. Most medical schools in Taiwan adopt definitions of the MH that have been developed by New York University, which defines MH as "an interdisciplinary field of humanities, social science, and the art and their application to healthcare education and practice [14] ".

### Study design and participants

We conducted a retrospective cohort analysis of students' data such as age, gender, residential area, weighted average mark, and clinical curriculum scores provided by the National Yang-Ming University register's office. The sample frame consisted of students admitted to the school of medicine of National Yang-Ming University in 2012, 2013, and 2014, as well as those who graduated from this university in 2018 and 2019 (*n* = 370). During the clerkship, all students worked mostly under the same rotating clinical specialties but with different rotation orders.

Most students were admitted based on results of the General Scholastic Ability Test (GSAT) and Advanced Subjects Test (AST), high-stakes college entrance tests for high school seniors held annually in January and July in Taiwan. To decrease the heterogeneity of study participants, we excluded 15 students admitted by other methods, such as the examination for overseas Chinese students. One student was also excluded due to the omission of demographic information. A final total of 354 students were included in the study. The study protocol was approved by the Institutional Review Board of Taipei Veterans General Hospital (2018–01-006CC).

### Study variables and outcomes

MHC was defined as curriculums of sustained interdisciplinary inquiry into aspects of medical practice, education, and research expressly concerned with the human side of medicine [15]. Independent variables included students' information such as residential area, gender, age at enrollment, type of administration, school program, the scores of MHC, and the adjusted number of MHC with better scores. Adjusted MHC number means when a student's MHC score is better than the median MHC score of all students, the participated MHC was then counted. We divided students into groups according to the median of MHC scores (above median group vs. below median group) and the adjusted MHC number (low MHC coursework group: 0–1 vs. high MHC coursework group:2–5).

The outcome variables assessed of this study included: 1). Clinical curriculum scores (cumulative scores for the third and fourth year), 2). Clerkship performance (cumulative scores for the fifth, sixth, and seventh years for the old school program, and the fifth and sixth years for the new school program), and 3). Weighted average mark.

The GSAT and AST are held separately by the College Entrance Examination Center annually in Taiwan. The main difference between the two tests is that the former includes preparing a portfolio and participating in an interview. At the same time, the latter is solely evaluated by exam scores to determine the distribution order to universities. It is also worth noting that in Taiwan, about 1/3–1/4 of medical students retake their exams to achieve their ideal school department, and most go to an entrance exam cram school to achieve their goal [16]. Few papers have researched the unique phenomenon of cram school fever in Taiwan. Thus, we were interested in exploring whether the two groups of students behaved differently in future performance in MHC. Besides, there is scarcely a concept of gap years after graduation from senior high school in Taiwan [17]. Therefore, As the cut-off point of the academic year in Taiwan is September 1^st^, we defined age > 18 with the date of birth before September 1^st^ when enrolling in medical school as resit students, and those aged younger than 18 years old were deemed to be new undergraduates. As for the school programs, students from both school programs were included. Furthermore, we divided students' residential areas into special municipalities and non-special municipalities. According to Taiwanese law, a city is considered a special municipality when the city's population is more than 1.25 million people and it has special needs in political, economic, cultural, and urban development.

### Statistical analyses

SAS version 9.4 (SAS Institute, Cary, NC, USA) was used for statistical analyses. Means and standard deviations were calculated for continuous variables. Percentages were calculated for nominal and ordinal variables. Descriptive statistical analyses, including the chi-squared test and student's t-test, were performed to examine the participants' demographic characteristics between groups. Multiple regression analyses were conducted after controlling for other potential confounders, such as gender, age at enrollment, school programs, residential area, and type of administration, to explore the relationship between adjusted MHC number and students' following performance. A two-sided *p*-value of < 0.05 was considered statistically significant.

## Results

There were 196 students in the above-median group compared with 158 students in the below-median group. More females were in the above-median group than in the below-median group (69% vs. 31%, *p* < 0.001). We found no statistically significant difference in the proportion living in a special municipality between the above-median group and the below-median group (54% vs. 46%, *p* = 0.31). In addition, fewer resit students were in the above-median group than in the below-median group (46% vs. 54%, *p* < 0.05). As for the distribution of the type of administration, in the above-median group, 73 students were admitted by GSAT, and 123 were admitted by AST, whereas GSAT admitted 78 students, and 80 were admitted by AST in the below-median group. Of the 244 seven-year medical students, 146 were in the above-median group, and 98 were in the below-median group. Among the 110 six-year medical students, 50 were in the above-median group, and 60 were in the below-median group. The average number of adjusted MHC students took in the above-median group was 2.06 ± 1.05, while that of the below-median group was 0.77 ± 0.69 (Table 1).

**Table 1:** Student characteristics in different subgroups

Variable	Below median group		Above median group		*P*-value	Low MHC coursework		High MHC coursework		*P*-value
	N	%	N	%		N	%	N	%	
Total	158		196			196		158		
Female	40	31%	90	69%	< 0.001	59	45%	71	55%	< 0.01
Special Municipality	131	46%	154	54%	0.31	159	56%	126	44%	0.75
Resit	43	54%	36	46%	< 0.05	47	59%	32	41%	0.40
Type of administration										
GSAT	78	0.22	73	0.21	< 0.05	84	0.24	67	0.19	0.93
AST	80	0.23	123	0.35		112	0.32	91	0.26	
School program										
6-year program	60	0.17	50	0.14	< 0.05	87	0.25	23	0.06	< 0.001
7-year program	98	0.28	146	0.41		109	0.31	135	0.38	
	Mean (SD)		Mean (SD)							
Adjusted number of humanity curriculums*	0.77	0.69	2.06	1.05						

*MHC* medical humanities curriculums

Above median group: students whose average score in the MHC was greater than the median class score in the MHC

Below median group: students whose average score in the MHC was lower than the median class score in the MHC

High MHC coursework: an adjusted number of 2–5 MHC

Low MHC coursework: an adjusted number of 0–1 MHC

^*^Adjusted number means when a student's MHC score is better than the median MHC score of all students, the MHC that was taken was counted

As high MHC coursework vs. low MHC coursework group, there were no statistically significant differences between the two groups regarding the place of residence (high urbanization), type of admission, and enrollment age. Nevertheless, 45% of females were in the low MHC coursework, while 55% of females were in the high MHC coursework (*p* < 0.01). A significant difference was also noted in the school programs between the low and high coursework groups (*p* < 0.001) (Table 1).

Students' performance, such as weighted average mark, clinical curriculum scores, and clerkship performance, was significantly higher in the above-median group when compared with the below-median group. (*p* < 0.001). Similar results were also observed between the high and low MHC coursework groups in weighted average mark and clerkship performance (*p* < 0.001). No significant difference was noted in clinical curriculum scores between the high and low MHC coursework groups (*p* = 0.13) (Table 2).

**Table 2:** Comparison of the correlation between learning quality and quantity of MHC with students’ performance in weighted average mark, clinical curriculum scores, and clerkship performance

	Below median group		Above median group		*P*-value	Low MHC coursework		High MHC coursework		*P*-value
	*N* = 158		*N* = 196			*N* = 196		*N* = 158		
	mean	SD	mean	SD		mean	SD	mean	SD	
Clinical curriculum scores	85.21	4.1	86.81	3.58	< 0.001	85.82	4.02	86.45	3.72	0.13
Clerkship performance	90.48	1.52	91.37	1.2	< 0.001	90.53	1.46	91.52	1.16	< 0.001
Weighted average mark	85.95	3.23	88.2	2.67	< 0.001	86.49	3.23	88.07	2.78	< 0.001

*MHC* medical humanities curriculums

Above median group: students whose average score in the MHC was greater than the median class score in the MHC

Below median group: students whose average score in the MHC was lower than the median class score in the MHC

High MHC coursework: an adjusted number* of 2–5 MHC

Low MHC coursework: an adjusted number* of 0–1 MHC

^*^Adjusted number means when a student's MHC score is better than the median MHC score of all students, the MHC that was taken was counted

Comparing students whose MHC scores were all lower than the median MHC score of the classes and students whose MHC scores were better than the median MHC score of the classes, the weighted average mark of students who took every additional adjusted number of MHC increased by 0.74 points (SE = 0.15, *p* < 0.001). Similarly, a positive correlation was also found between clinical curriculum scores and clerkship performance [β = 0.38, (SE = 0.20 *p* < 0.05) and β = 0.31 (SE = 0.07, *p* < 0.001), respectively) (Table 3). Among other independent variables, males had the significantly lower weighted average mark, clinical curriculum scores, and clerkship performance (β = -1.53, -1.47, -0.75; all *p* < 0.001). New graduates had lower scores than resit in weighted average mark and clinical curriculum scores (β = -1.7, -2.16, all *p* < 0.001), and there were statistically significant differences in weighted average mark and clinical curriculum scores (both *p* < 0.001), but not in clerkship performance (*p* = 0.07). Moreover, in terms of school programs, six-year students performed better in weighted average mark and clinical curriculum scores, while seven-year students performed better in clerkship (all *p* < 0.01). Finally, residential area and type of administration had no statistically significant effect on students' performances (Table 3).

**Table 3:** Multivariable regression analyses for the relationship between the adjusted number of MHC and students’ following performance

	Clinical curriculum scores			Clerkship performance			Weighted average mark		
	β	SE	p	β	SE	p	β	SE	p
Intercept	88.34	0.96	< 0.001	90.11	0.33	< 0.001	87.71	0.75	< 0.001
Number of adjusted humanity curriculums									
	0.38	0.20	< 0.05	0.31	0.07	< 0.001	0.74	0.15	< 0.001
Gender									
female									
male	-1.47	0.41	< 0.001	-0.75	0.14	< 0.001	-1.53	0.32	< 0.001
Residential area									
Special Municipality									
Non special municipality	0.39	0.49	0.42	0.11	0.168	0.50	0.18	0.38	0.65
Age at enrollment									
Resit									
New graduate	-2.16	0.47	< 0.001	-0.3	0.16	0.07	-1.70	0.37	< 0.001
Type of administration									
GSAT									
AST	-0.07	0.40	0.87	0.27	0.14	0.05	0.18	0.32	0.57
School program									
6-year program									
7-year program	-2.33	0.46	< 0.001	0.61	0.16	< 0.001	-1.01	0.36	< 0.01

*MHC* medical humanities curriculums, *GSAT* General Scholastic Ability Test, *AST* Advanced Subjects Test

**Fig. 1:**
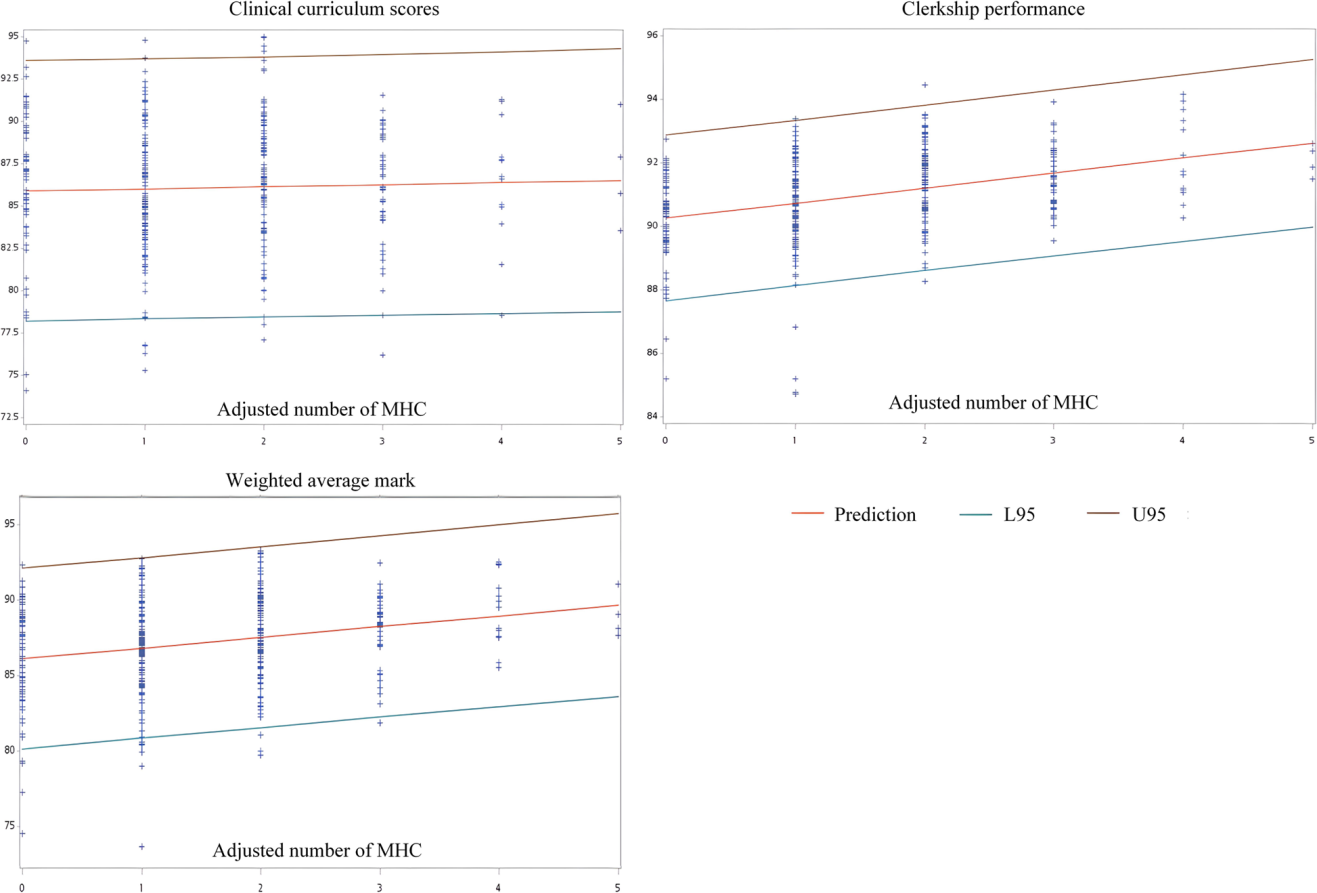
The correlation between the adjusted number of MHC and students' performance (scores: clinical curriculum scores, clerkship performance, and weighted average mark). MHC: medical humanities curriculums   Adjusted number of MHC: when a student's MHC score is better than the median MHC score of all students, the MHC that was taken was counted

Figure 1 shows that the adjusted number of MHC was positively correlated with students' performance (clinical curriculum scores, clerkship performance, and weighted average mark).

## Discussion

In the current study, we demonstrated that students' performance in MH and the number of MHC taken by students were positively associated with clinical curriculum scores, clerkship performance, and weighted average mark. To the best of our knowledge, this study is the first to predict medical students' academic and clinical performance by quantifying MHC in Asia. We found that compared with the number of MHC, the learning quality of MHC showed a more significant correlation with medical students' clinical performance and weighted average mark.

Previous studies using questionnaires or online surveys have found the positive influence of MH on certain characteristics, such as empathy [18, 19]. An article conducted at Harvard University also quantified the performance of the MH course and obtained results consistent with our study, which showed linear relationships between the humanistic medicine course and clinical performance (*p* = 0.03) [10]. Another study also conducted quantitative analysis on fourth-year medical students in the United States [20]. It reached similar conclusions that students in the humanities curriculums showed statistically significant and educationally meaningful gains in communication skills compared with the control group [20]. The result of a 2019 study on the impact of MH courses on clinical competence, measured through objective structured clinical examinations, is also in line with our research [21]. On the other hand, a randomized controlled trial published in 2020 evaluated the potential benefits of integrating MHC into clerkship courses [22]. Nevertheless, it showed no observable quantitative differences in tracked National Board of Medical Examiners shelf exam performance [22]. The conflicting result may be explained by relatively little exposure to MHC, a total contact time of 3 h for the intervention group [22].

However, the studies mentioned above have their limitations, whether they focus on analyzing a single MH course or questionnaire-based research, which may have potential bias [23]. Consequently, those studies on the impact of MHC inevitably lack robust, objective, and measurable evidence. In the present study, instead, we analyzed objective MHC scores with more generalizable and more comprehensive methods and found that MH education positively correlated with clerkship performance and weighted average mark.

Unlike most previous articles, which praised the (potential) impact of the humanities on medical education in a single curriculum or in a more subjective manner [24], we further quantified the correlation between the adjusted number of MHC and the students’ following performance. Besides, in supplementary table 1, we also found that students who take more than five MHC performed worse than those who take fewer MHC. That is to say: our research has further proved that it is not "more is better" in MHC. The positive correlation between MH education and medical students’ following performance can be fully reflected only when both the quality of curriculums and the number of appropriate curriculums are taken into consideration. The possible reason why medical students' performance is not proportional to the number of MHC may be due to tight study schedules; learners feel constant pressure to excel under the specter of constant evaluation [25]. This is a cause of concern as the high prevalence of stress among medical students may impair the behavior of students, diminish learning, and ultimately affect patient care after their graduation.

It is noteworthy that no correlation between the adjusted number of humanities curriculums and clinical curriculum scores, assessed by written examination, was observed. Combined with the fact that the performance of clerkship was better in the group with a higher adjusted number of MHC, this finding indicates that MHC had greater influence on clinical performance. Some studies support our results. Gordon et al. showed that MH could overcome the separation of clinical care from the "human sciences" and foster interdisciplinary teaching to optimize patient care through a more holistic approach [26]. Moreover, diverse MHC, such as arts and sports activities, could help to prepare students for challenging cases and lectures [27, 28]. Another report revealed that taking MHC may provide much-needed opportunities for self-reflection about the intensive process of becoming a physician and may ease feelings of isolation or burnout [29]. This is essential as it has been shown that burnout is a significant challenge during the early training years of residency and is influenced by time demands, lack of control work planning, work environment and setting, inherently difficult job situations, and interpersonal relationships [30].

In the present study, we also evaluated the correlation between different confounding factors, such as gender, residential area, age at enrollment, type of administration, school program with our outcomes. Our results showed that the male gender was a negative predictor for weighted average mark, clinical curriculum scores, and clerkship performance. This may be explained by higher self-efficacy in males than in females, which has been reported previously in medical students [31, 32]. Furthermore, several studies also concluded that females performed better than their male counterparts in interpersonal skills and were more patient-centered [33, 34]. The length of the medical school curriculum in Taiwan has been shortened from seven to six years since 2013 [11]. In addition to strengthening clinical training and improving educational outcomes based on the concept of "learning-by-doing", the curricular reform is also relevant to the gray area of implementing medical interventions during clerkships. It is interesting to note that students who received seven-year medical education had a better performance in clerkship performance when compared with six-year medical students. The better performance in clerkship may be due to more experience of patient care and clinical practice, and may also be related to the better performance in MHC in seven-year students, as shown in Table 1. However, the shorter medical school program course may not seem to interfere with students' clinical performance development. A study conducted in Taiwan demonstrated that medical students studying a six-year program had lower objective structured clinical examination (OSCE) scores at the beginning of their clerkship, but there were no differences between six- and seven-year program in OSCE performances after the clerkship training [35]. Moreover, our results did not show a significant difference when a residential area was included as a confounding factor in a student's performance. This result may be explained by the fact that Taiwan is a highly urbanized country, and the gap between urban and rural areas is not especially obvious [36].

Our study had several limitations. First, the sample comprised only one cohort of medical students from a single medical school in Taiwan, which may limit the generalizability of our results. Second, some scholars argue that humanities performance cannot be quantified numerically [37]. Consequently, we should interpret our results carefully since MH education focuses on the process of establishing self-values, and it takes time to develop and internalize these concepts. Therefore, our assessment time points may not be optimal. Third, this article does not address the correlation between MHC and postgraduate medical students, such as PGY or residency performance. Caution is required when inferring final impact after graduation. Fourth, since many MHCs are elective curriculums, there was inevitably some potential selection bias in this study. Nevertheless, it is worth noting that some MHCs were already included as required curriculums in the recent curriculum reform. However, it is impossible to create a randomized controlled trial for comparing differences between MHC due to ethical issues. A more in-depth exploration to identify possible relevant humanities curriculums is warranted in future research. Fifth, since our definition of resit is based on the time of admission and age, the resit group may include some non-repeaters, such as gap year or grade retention. Further coordinated, multi-institutional study is needed to more firmly establish the appropriate role of the MH in modern medical education.

## Conclusion

By controlling for various confounding factors, such as gender, enrollment methods, and differences in schooling programs, our study quantifies the correlation between academic performance and MHC for medical students. Our experience may be of benefit to medical educators and could inspire future curriculum reforms in medical schools. We recommend a future study of improving the quality of MH education to see if this contributes to student learning outcomes.

### Supplementary Information

**Additional file 1.**

## Data Availability

The datasets used and/or analysed during the current study are available from the corresponding author on reasonable request.

## Abbreviations

MH: Medical Humanities
MHC: Medical Humanities Curriculums
GSAT: General Scholastic Ability Test
AST: Advanced Subjects Test
OSCE: Objective structured clinical examination
