# Supplementary material for: The quantified analysis of the correlation between medical humanities curriculums and medical students’ performance
Source: BMC Med Educ. 2023 Aug 11;23:571. doi: 10.1186/s12909-023-04073-y (PMC10422819; doi:10.1186/s12909-023-04073-y)
Supplement: Supplementary file 1 — Additional file 1. [file 12909_2023_4073_MOESM1_ESM.docx]

Supplementary table 1. Comparison of the correlation between different amounts of MHC and students’ following performance

|  | Number of MHC  (0-3)  N=114 | | Number of MHC  (4)  N=134 | | Number of MHC  (5-8)  N=106 | | P value |
| --- | --- | --- | --- | --- | --- | --- | --- |
|  | Mean | SD | Mean | SD | Mean | SD |  |
| Clinical curriculum scores | 86.76 | 3.98 | 86.17 | 3.69 | 85.30 | 3.95 | <0.05 |
| Clerkship performance | 90.97 | 1.26 | 91.07 | 1.26 | 90.85 | 1.62 | 0.50 |
| Weighted Average Mark | 87.47 | 3.16 | 87.50 | 2.70 | 86.51 | 3.52 | <0.05 |

MHC: medical humanities curriculums
